# Death of a Hospital Founder

**Published:** 1924-05

**Authors:** 


### Death of a Hospital Founder.

Mr. William Carrington-Smith, who has died at the age of 75, was the founder of the Acton (Middlesex) Hospital, and, before he retired in 1920, had held the office of president for 22 years.

